# pROC: an open-source package for R and S+ to analyze and compare ROC curves

**DOI:** 10.1186/1471-2105-12-77

**Published:** 2011-03-17

**Authors:** Xavier Robin, Natacha Turck, Alexandre Hainard, Natalia Tiberti, Frédérique Lisacek, Jean-Charles Sanchez, Markus Müller

**Affiliations:** 1 Biomedical Proteomics Research Group, Department of Structural Biology and Bioinformatics, Medical University Centre, Geneva, Switzerland; 2 Swiss Institute of Bioinformatics, Medical University Centre, Geneva, Switzerland

## Abstract

**Background:**

Receiver operating characteristic (ROC) curves are useful tools to evaluate classifiers in biomedical and bioinformatics applications. However, conclusions are often reached through inconsistent use or insufficient statistical analysis. To support researchers in their ROC curves analysis we developed *pROC*, a package for R and S+ that contains a set of tools displaying, analyzing, smoothing and comparing ROC curves in a user-friendly, object-oriented and flexible interface.

**Results:**

With data previously imported into the R or S+ environment, the *pROC *package builds ROC curves and includes functions for computing confidence intervals, statistical tests for comparing total or partial area under the curve or the operating points of different classifiers, and methods for smoothing ROC curves. Intermediary and final results are visualised in user-friendly interfaces. A case study based on published clinical and biomarker data shows how to perform a typical ROC analysis with *pROC*.

**Conclusions:**

*pROC *is a package for R and S+ specifically dedicated to ROC analysis. It proposes multiple statistical tests to compare ROC curves, and in particular partial areas under the curve, allowing proper ROC interpretation. *pROC *is available in two versions: in the R programming language or with a graphical user interface in the S+ statistical software. It is accessible at http://expasy.org/tools/pROC/ under the GNU General Public License. It is also distributed through the CRAN and CSAN public repositories, facilitating its installation.

## Background

A ROC plot displays the performance of a binary classification method with continuous or discrete ordinal output. It shows the sensitivity (the proportion of correctly classified positive observations) and specificity (the proportion of correctly classified negative observations) as the output threshold is moved over the range of all possible values. ROC curves do not depend on class probabilities, facilitating their interpretation and comparison across different data sets. Originally invented for the detection of radar signals, they were soon applied to psychology [1] and medical fields such as radiology [2]. They are now commonly used in medical decision making, bioinformatics[3], data mining and machine learning, evaluating biomarker performances or comparing scoring methods [2,4].

In the ROC context, the area under the curve (AUC) measures the performance of a classifier and is frequently applied for method comparison. A higher AUC means a better classification. However, comparison between AUCs is often performed without a proper statistical analysis partially due to the lack of relevant, accessible and easy-to-use tools providing such tests. Small differences in AUCs can be significant if ROC curves are strongly correlated, and without statistical testing two AUCs can be incorrectly labelled as similar. In contrast a larger difference can be non significant in small samples, as shown by Hanczar *et al. *[5], who also provide an analytical expression for the variance of AUC's as a function of the sample size. We recently identified this lack of proper statistical comparison as a potential cause for the poor acceptance of biomarkers as diagnostic tools in medical applications [6]. Evaluating a classifier by means of total AUC is not suitable when the performance assessment only takes place in high specificity or high sensitivity regions [6]. To account for these cases, the partial AUC (pAUC) was introduced as a local comparative approach that focuses only on a portion of the ROC curve [7-9].

Software for ROC analysis already exists. A previous review [10] compared eight ROC programs and found that there is a need for a tool performing valid and standardized statistical tests with good data import and plot functions.

The R [11] and S+ (TIBCO Spotfire S+ 8.2, 2010, Palo Alto, CA) statistical environments provide an extensible framework upon which software can be built. No ROC tool is implemented in S+ yet while four R packages computing ROC curves are available:

1) *ROCR *[12] provides tools computing the performance of predictions by means of precision/recall plots, lift charts, cost curves as well as ROC plots and AUCs. Confidence intervals (CI) are supported for ROC analysis but the user must supply the bootstrapped curves.

2) The *verification *package [13] is not specifically aimed at ROC analysis; nonetheless it can plot ROC curves, compute the AUC and smooth a ROC curve with the binomial model. A Wilcoxon test for a single ROC curve is also implemented, but no test comparing two ROC curves is included.

3) Bioconductor includes the *ROC *package [14] which can only compute the AUC and plot the ROC curve.

4) Pcvsuite [15] is an advanced package for ROC curves which features advanced functions such as covariate adjustment and ROC regression. It was originally designed for Stata and ported to R. It is not available on the CRAN (comprehensive R archive network), but can be downloaded for Windows and MacOS from http://labs.fhcrc.org/pepe/dabs/rocbasic.html.

Table 1 summarizes the differences between these packages. Only pcvsuite enables the statistical comparison between two ROC curves. Pcvsuite, ROCR and ROC can compute AUC or pAUC, but the pAUC can only be defined as a portion of specificity.

**Table 1:** Features of the R packages for ROC anaylsis

Package name	ROCR	Verification	ROC (Bioconductor)	pcvsuite	pROC
Smoothing	No	Yes	No	Yes	Yes

Partial AUC	Only SP^1^	No	Only SP^1^	Only SP	SP and SE

Confidence intervals	Partial^2^	Partial^3^	No	Partial^4^	Yes

Plotting Confidence Intervals	Yes	Yes	No	Yes	Yes

Statistical tests	No	AUC (one sample)	No	AUC, pAUC, SP	AUC, pAUC, SP, SE, ROC

Available on CRAN	Yes	Yes	No, http://www.bioconductor.org/	No, http://labs.fhcrc.org/pepe/dabs/	Yes

^1^Partial AUC only between 100% and a specified cutoff of specificity

^2^Bootstrapped ROC curves must be computed by the user

^3^Only threshold averaging

^4^Only at a given specificity or inverse ROC

The *pROC *package was designed in order to facilitate ROC curve analysis and apply proper statistical tests for their comparison. It provides a consistent and user-friendly set of functions building and plotting a ROC curve, several methods smoothing the curve, computing the full or partial AUC over any range of specificity or sensitivity, as well as computing and visualizing various CIs. It includes tests for the statistical comparison of two ROC curves as well as their AUCs and pAUCs. The software comes with an extensive documentation and relies on the underlying R and S+ systems for data input and plots. Finally, a graphical user interface (GUI) was developed for S+ for users unfamiliar with programming.

## Implementation

### AUC and pAUC

In *pROC*, the ROC curves are empirical curves in the sensitivity and specificity space. AUCs are computed with trapezoids [4]. The method is extended for pAUCs by ignoring trapezoids outside the partial range and adding partial trapezoids with linear interpolation when necessary. The pAUC region can be defined either as a portion of specificity, as originally described by McClish [7], or as a portion of sensitivity, as proposed later by Jiang *et al. *[8]. Any section of the curve pAUC(t_0_, t_1_) can be analyzed, and not only portions anchored at 100% specificity or 100% sensitivity. Optionally, pAUC can be standardized with the formula by McClish [7]:(1)

where *min *is the pAUC over the same region of the diagonal ROC curve, and *max *is the pAUC over the same region of the perfect ROC curve. The result is a standardized pAUC which is always 1 for a perfect ROC curve and 0.5 for a non-discriminant ROC curve, whatever the partial region defined.

### Comparison

Two ROC curves are "paired" (or sometimes termed "correlated" in the literature) if they derive from multiple measurements on the same sample. Several tests exist to compare paired [16-22] or unpaired [23] ROC curves. The comparison can be based on AUC [16-19,21], ROC shape [20,22,23], a given specificity [15] or confidence bands [3,24]. Several tests are implemented in *pROC*. Three of them are implemented without modification from the literature [17,20,23], and the others are based on the bootstrap percentile method.

The bootstrap test to compare AUC or pAUC in *pROC *implements the method originally described by Hanley and McNeil [16]. They define Z as(2)

where *θ*_1 _and *θ*_2 _are the two (partial) AUCs. Unlike Hanley and McNeil, we compute *sd*(*θ*_1 _- *θ*_2_) with N (defaults to 2000) bootstrap replicates. In each replicate *r*, the original measurements are resampled with replacement; both new ROC curves corresponding to this new sample are built, the resampled AUCs *θ*_1,*r *_and *θ*_2,*r *_and their difference *D_r _*= *θ*_1,*r *_- *θ*_2,*r *_are computed. Finally, we compute *sd*(*θ*_1 _- *θ*_2_) = *sd*(*D*). As Z approximately follows a normal distribution, one or two-tailed p-values are calculated accordingly. This bootstrap test is very flexible and can be applied to AUC, pAUC and smoothed ROC curves.

Bootstrap is stratified by default; in this case the same number of case and control observations than in the original sample will be selected in each bootstrap replicate. Stratification can be disabled and observations will be resampled regardless of their class labels. Repeats for the bootstrap and progress bars are handled by the *plyr *package [25].

The second method to compare AUCs implemented in *pROC *was developed by DeLong et al. [17] based on U-statistics theory and asymptotic normality. As this test does not require bootstrapping, it runs significantly faster, but it cannot handle pAUC or smoothed ROC curves. For both tests, since the variance depends on the covariance of the ROC curves (Equation 3), strongly correlated ROC curves can have similar AUC values and still be significantly different.(3)

Venkatraman and Begg [20] and Venkatraman [23] introduced tests to compare two actual ROC curves as opposed to their respective AUCs. Their method evaluates the integrated absolute difference between the two ROC curves, and a permutation distribution is generated to compute the statistical significance of this difference. As the measurements leading to the two ROC curves may be performed on different scales, they are not generally exchangeable between two samples. Therefore, the permutations are based on ranks, and ranks are recomputed as described in [20] to break the ties generated by the permutation.

Finally a test based on bootstrap is implemented to compare the ROC curve at a given level of specificity or sensitivity as proposed by Pepe *et al. *[15]. It works similar to the (p)AUC test, but instead of computing the (p)AUC at each iteration, the sensitivity (or specificity) corresponding to the given specificity (or respectively sensitivity) is computed. This test is equivalent to a pAUC test with a very small pAUC range.

### Confidence intervals

CIs are computed with Delong's method [17] for AUCs and with bootstrap for pAUCs [26]. The CIs of the thresholds or the sensitivity and specificity values are computed with bootstrap resampling and the averaging methods described by Fawcett [4]. In all bootstrap CIs, patients are resampled and the modified curve is built before the statistics of interest is computed. As in the bootstrap comparison test, the resampling is done in a stratified manner by default.

### Smoothing

Several methods to smooth a ROC curve are also implemented. Binormal smoothing relies on the assumption that there exists a monotone transformation to make both case and control values normally distributed [2]. Under this condition a simple linear relationship (Equation 4) holds between the normal quantile function (*φ*) values of sensitivities and specificities. In our implementation, a linear regression between all quantile values defines *a *and *b*, which then define the smoothed curve.(4)

This is different from the method described by Metz et al. [27] who use maximum likelihood estimation of *a *and *b*. Binormal smoothing was previously shown to be robust and to provide good fits in many situations even when the deviation from basic assumptions is quite strong [28]. For continuous data we also include methods for kernel (density) smoothing [29], or to fit various known distributions to the class densities with *fitdistr *in the MASS package [30]. If a user would like to run a custom smoothing algorithm that is optimized for the analysed data, then *pROC *also accepts class densities or the customized smoothing function as input. CI and statistical tests of smoothed AUCs are done with bootstrap.

## Results and Discussion

We first evaluate the accuracy of the ROC comparison tests. Results in Additional File 1 show that all unpaired tests give uniform p-values under a null hypothesis (Additional Files 1 and 2) and that there is a very good correlation between DeLong's and bootstrap tests (Additional Files 1 and 3). The relation between Venkatraman's and the other tests is also investigated (Additional Files 1 and 4).

We now present how to perform a typical ROC analysis with *pROC*. In a recent study [31], we analyzed the level of several biomarkers in the blood of patients at hospital admission after aneurysmal subarachnoid haemorrhage (aSAH) to predict the 6-month outcome. The 141 patients collected were classified according to their outcome with a standard neurological scale, the Glasgow outcome scale (GOS). The biomarker performances were compared with the well established neurological scale of the World Federation of Neurological Surgeons (WFNS), also obtained at admission.

### Case study on clinical aSAH data

The purpose of the case presented here is to identify patients at risk of poor post-aSAH outcome, as they require specific healthcare management; therefore the clinical test must be highly specific. Detailed results of the study are reported in [31]. We only outline the features relevant to the ROC analysis.

ROC curves were generated in *pROC *for five biomarkers (H-FABP, S100β, Troponin I, NKDA and UFD-1) and three clinical factors (WFNS, Modified Fisher score and age).

#### AUC and pAUC

Since we are interested in a clinical test with a high specificity, we focused on partial AUC between 90% and 100% specificity.

**Figure 1:**
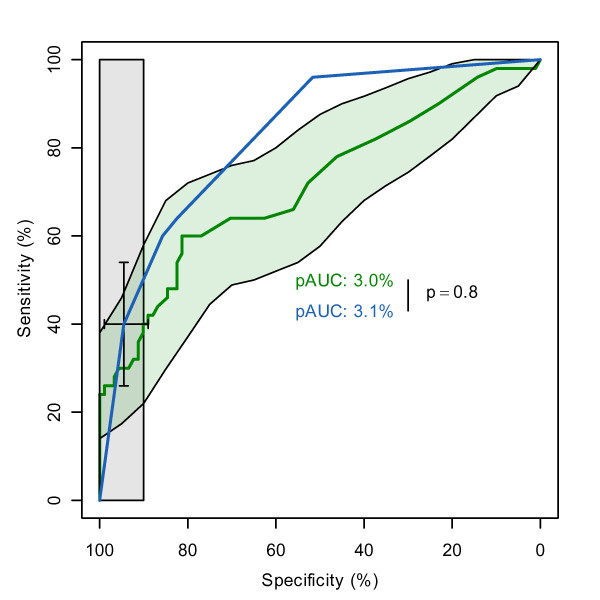
**ROC curves of WFNS and S100β**. ROC curves of WFNS (blue) and S100β (green). The black bars are the confidence intervals of WFNS for the threshold 4.5 and the light green area is the confidence interval shape of S100β. The vertical light grey shape corresponds to the pAUC region. The pAUC of both empirical curves is printed in the middle of the plot, with the p-value of the difference computed by a bootstrap test on the right.

The best pAUC is obtained by WFNS, with 3.1%, closely followed by S100β with 3.0% (Figure 1). A perfect clinical test within the same region corresponds to a pAUC of 10%, while a ROC curve without any discrimination power would yield only 0.5%. In the case of WFNS, we computed a standardized pAUC of 63.7% with McClish's formula (Equation 1). Of these 63.9%, 50% are due to the small portion (0.5% non-standardized) of the ROC curve below the identity line, and the remaining 13.9% are made of the larger part (2.6% non-standardized) above the curve. In the R version of *pROC*, the standardized pAUC of WFNS can be computed with:

roc(response = aSAH$outcome, predictor = aSAH$wfns, partial.auc = c(100, 90), partial.auc.correct = TRUE, percent = TRUE)

In the rest of this paper, we report only not standardized pAUCs.

#### CI

Given the pAUC of WFNS, it makes sense to compute a 95% CI of the pAUC to assess the variability of the measure. In this case, we performed 10000 bootstrap replicates and obtained the 1.6-5.0% interval. In our experience, 10000 replicates give a fair estimate of the second significant digit. A lower number of replicates (for example 2000, the default) gives a good estimate of the first significant digit only. Other confidence intervals can be computed. The threshold with the point farthest to the diagonal line in the specified region was determined with pROC to be 4.5 with the *coords *function. A rectangular confidence interval can be computed and the bounds are 89.0-98.9 in specificity and 26.0-54.0 in sensitivity (Figure 1). If the variability of sensitivity at 90% specificity is considered more relevant than at a specific threshold, the interval of sensitivity is computed as 32.8-68.8. As shown in Figure 1 for S100β, a CI shape can be obtained by simply computing the CI's of the sensitivities over several constantly spaced levels of specificity, and these CI bounds are then joined to generate the shape. The following R code calculates the confidence shape:

plot(roc = roc(response = aSAH$outcome, predictor = aSAH$s100, percent = TRUE, ci = TRUE, of = "se", sp = seq(0, 100, 5)), ci.type="shape")

The confidence intervals of a threshold or of a predefined level of sensitivity or specificity answer different questions. For instance, it would be wrong to compute the CI of the threshold 4.5 and report only the CI bound of sensitivity without reporting the CI bound of specificity as well. Similarly, determining the sensitivity and specificity of the cut-off 4.5 and then computing both CIs separately would also be inaccurate.

#### Statistical comparison

The second best pAUC is that of S100β with 3.0%. The difference to WFNS is very small and the bootstrap test of *pROC *indicates that it is not significant (p = 0.8, Figure 1). Surprisingly, a Venkatraman's test (over the total ROC curve) indicates a difference in the shape of the ROC curves (p = 0.004), and indeed a test evaluating pAUCs in the high sensitivity region (90-100% sensitivity) would highlight a significant difference (p = 0.005, pAUC = 4.3 and 1.4 for WFNS and S100β respectively). However, since we are not interested in the high sensitivity region of the AUC there is no significant difference between WFNS and S100β.

In *pROC *pairwise comparison of ROC curves is implemented. Multiple testing is not accounted for and in the event of running several tests, the user is reminded that as with any statistical test, multiple tests should be performed with care, and if necessary appropriate corrections should be applied [32].

The bootstrap test can be performed with the following code in R:

roc.test(response = aSAH$outcome, predictor1 = aSAH$wfns, predictor2 = aSAH$s100, partial.auc = c(100, 90), percent = TRUE)

#### Smoothing

**Figure 2:**
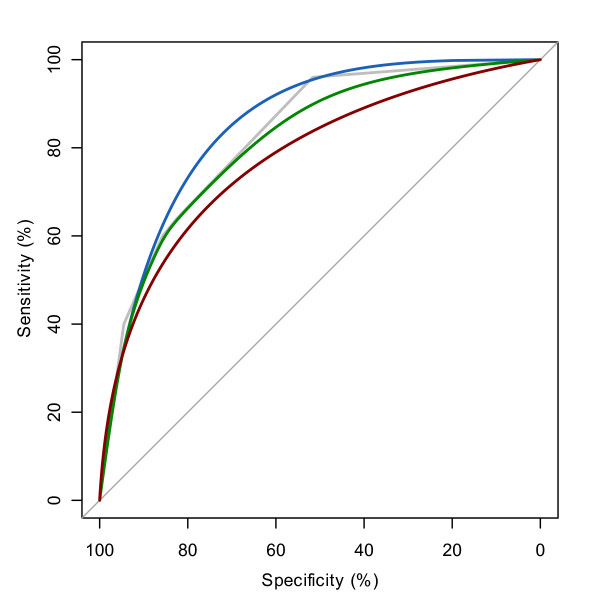
**ROC curve of WFNS and smoothing**. Empirical ROC curve of WFNS is shown in grey with three smoothing methods: binormal (blue), density (green) and normal distribution fit (red).

Whether or not to smooth a ROC curve is a difficult choice. It can be useful in ROC curves with only few points, in which the trapezoidal rule consistently underestimates the true AUC [17]. This is the case with most clinical scores, such as the WFNS shown in Figure 2 where three smoothing methods available in *pROC *are plotted: (i) normal distribution fitting, (ii) density and (iii) binormal. In our case study:

(i) The normal fitting (red) gives a significantly lower AUC estimate (Δ = -5.1, p = 0.0006, Bootstrap test). This difference is due to the non-normality of WFNS. Distribution fitting can be very powerful when there is a clear knowledge of the underlying distributions, but should be avoided in other contexts.

(ii) The density (green) smoothing also produces a lower (Δ = -1.5, p = 6*10^-7^) AUC. It is interesting to note that even with a smaller difference in AUCs, the p-value can be more significant due to a higher covariance.

(iii) The binormal smoothing (blue) gives a slightly but not significantly higher AUC than the empirical ROC curve (Δ = +2.4, p = 0.3). It is probably the best of the 3 smoothing estimates in this case (as mentioned earlier we were expecting a higher AUC as the empirical AUC of WFNS was underestimated). For comparison, Additional File 5 displays both our implementation of binormal smoothing with the one implemented in pcvsuite [15].

**Figure 3:**
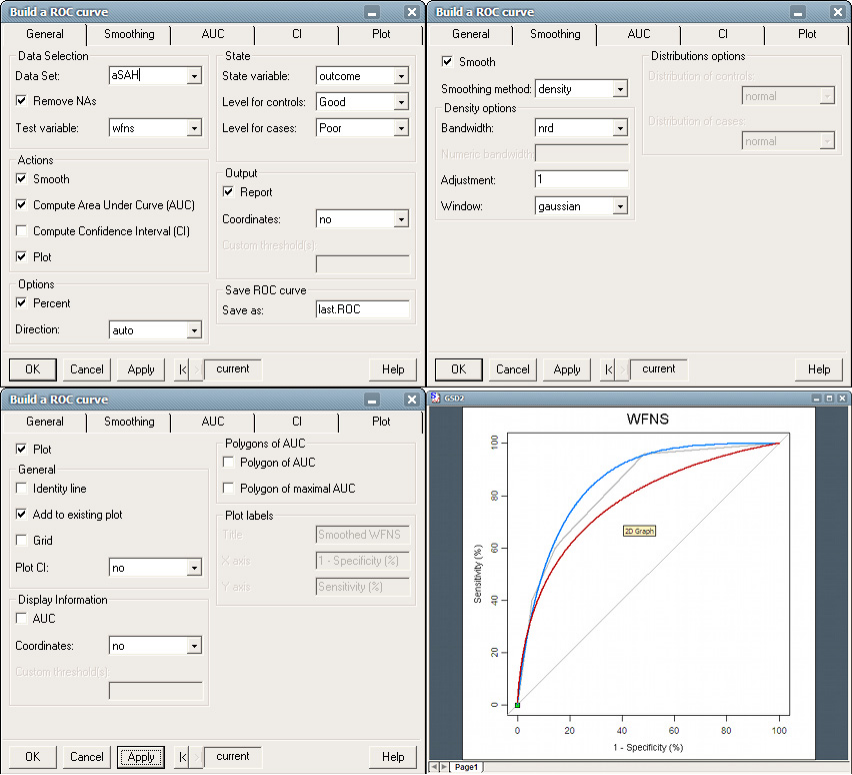
**Screenshot of *pROC *in S+ for smoothing WFNS ROC curve**. Top left: the General tab, where data is entered. Top right: the details about smoothing. Bottom left: the details for the plot. Checking the box "Add to existing plot" allows drawing several curves on a plot. Bottom right: the result in the standard S+ plot device.

Figure 3 shows how to create a plot with multiple smoothed curves with *pROC *in S+. One loads the pROC library within S+, selects the new *ROC curve *item in the S*tatistics *menu, selects the data on which the analysis is to be performed, and then moves to the *Smoothing *tab to set parameters for smoothing.

#### Conclusion

In this case study we showed how *pROC *could be run for ROC analysis. The main conclusion drawn from this analysis is that none of the measured biomarkers can predict the patient outcome better than the neurological score (WFNS).

### Installation and usage

#### R

*pROC *can be installed in R by issuing the following command in the prompt:

install.packages("pROC")

Loading the package:

library(pROC)

Getting help:

?pROC

#### S+

*pROC *is available from the *File *menu, item *Find Packages...*. It can be loaded from the *File *menu, item *Load Library....*

In addition to the command line functions, a GUI is then available in the *Statistics *menu. It features one window for univariate ROC curves (which contains options for smoothing, pAUC, CIs and plotting) and two windows for paired and unpaired tests of two ROC curves. In addition a specific help file for the GUI is available from the same menu.

### Functions and methods

A summary of the functions available to the user in the command line version of pROC is shown in Table 2. Table 3 shows the list of the methods provided for plotting and printing.

**Table 2:** Functions provided in pROC

are.paired	Determines if two ROC curves are possibly paired
auc	Computes the area under the ROC curve

ci	Computes the confidence interval of a ROC curve

ci.auc	Computes the confidence interval of the AUC

ci.se	Computes the confidence interval of sensitivities at given specificities

ci.sp	Computes the confidence interval of specificities at given sensitivities

ci.thresholds	Computes the confidence interval of thresholds

coords	Returns the coordinates (sensitivities, specificities, thresholds) of a ROC curve

roc	Builds a ROC curve

roc.test	Compares the AUC of two correlated ROC curves

smooth	Smoothes a ROC curve

**Table 3:** Methods provided by pROC for standard functions

lines	ROC curves (roc) and smoothed ROC curves (smooth.roc)
plot	ROC curves (roc), smoothed ROC curves (smooth.roc) and confidence intervals (ci.se, ci.sp, ci.thresholds)

print	All pROC objects (auc, ci.auc, ci.se, ci.sp, ci.thresholds, roc, smooth.roc)

## Conclusions

The *pROC *package is a powerful set of tools analyzing and comparing ROC curves in R and S+. Unlike existing packages such as *ROCR *or *verification*, it is solely dedicated to ROC analysis, but provides in our knowledge the most complete set of statistical tests and plots for ROC curves. As shown in the case study reported here, *pROC *features the computation of AUC and pAUC, various kinds of confidence intervals, several smoothing methods, and the comparison of two paired or unpaired ROC curves. We believe that *pROC *should provide researchers, especially in the biomarker community, with the necessary tools to better interpret their results in biomarker classification studies.

*pROC *is available in two versions for R and S+. A thorough documentation with numerous examples is provided in the standard R format. For users unfamiliar with programming, a graphical user interface is provided for S+.

## Availability and requirements

• Project name: pROC

• Project home page: http://expasy.org/tools/pROC/

• Operating system(s): Platform independent

• Programming language: R and S+

• Other requirements: R ≥ 2.10.0 or S+ ≥ 8.1.1

• License: GNU GPL

• Any restrictions to use by non-academics: none

## List of abbreviations

aSAH: aneurysmal subarachnoid haemorrhage; AUC: area under the curve; CI: confidence interval; CRAN: comprehensive R archive network; CSAN: comprehensive S-PLUS archive network; pAUC: partial area under the curve; ROC: receiver operating characteristic.

## Authors' contributions

XR carried out the programming and software design and drafted the manuscript. NTu, AH, NTi provided data and biological knowledge, tested and critically reviewed the software and the manuscript. FL helped to draft and to critically improve the manuscript. JCS conceived the biomarker study, participated in its design and coordination, and helped to draft the manuscript. MM participated in the design and coordination of the bioinformatics part of the study, participated in the programming and software design and helped to draft the manuscript. All authors read and approved the final manuscript.

## Supplementary Material

Additional file 1 **Assessment of the ROC comparison tests**. We evaluate the uniformity of the tests under the null hypothesis (ROC curves are not different), and the correlation between the different tests. Click here for file

Additional file 2 **Histograms of the frequency of 600 test p-values under the null hypothesis (ROC curves are not different)**. A: DeLong's paired test, B: DeLong's unpaired test, C: bootstrap paired test (with 10000 replicates), D: bootstrap unpaired test (with 10000 replicates) and E: Venkatraman's test (with 10000 permutations). Click here for file

Additional file 3 **Correlations between DeLong and bootstrap paired tests**. X axis: DeLong's test; Y-axis: bootstrap test with number of bootstrap replicates. A: 10, B: 100, C: 1000 and D: 10000. Click here for file

Additional file 4 **Correlation between DeLong and Venkatraman's test**. X axis: DeLong's test; Y-axis: Venkatraman's test with 10000 permutations. Click here for file

Additional file 5 **Binormal smoothing**. Binormal smoothing with pcvsuite (green, solid) and pROC (black, dashed). Click here for file

## References

[B1] Swets JA The Relative Operating Characteristic in Psychology Science 1973 182 990 1000 10.1126/science.182.4116.990 17833780

[B2] Pepe MS The statistical evaluation of medical tests for classification and prediction 2003 Oxford: Oxford University Press

[B3] Sonego P Kocsor A Pongor S ROC analysis: applications to the classification of biological sequences and 3D structures Brief Bioinform 2008 9 198 209 10.1093/bib/bbm064 18192302

[B4] Fawcett T An introduction to ROC analysis Pattern Recogn Lett 2006 27 861 874 10.1016/j.patrec.2005.10.010

[B5] Hanczar B Hua J Sima C Weinstein J Bittner M Dougherty ER Small-sample precision of ROC-related estimates Bioinformatics 2010 26 822 830 10.1093/bioinformatics/btq037 20130029

[B6] Robin X Turck N Hainard A Lisacek F Sanchez JC Müller M Bioinformatics for protein biomarker panel classification: What is needed to bring biomarker panels into in vitro diagnostics? Expert Rev Proteomics 2009 6 675 689 10.1586/epr.09.83 19929612

[B7] McClish DK Analyzing a Portion of the ROC Curve Med Decis Making 1989 9 190 195 10.1177/0272989X8900900307 2668680

[B8] Jiang Y Metz CE Nishikawa RM A receiver operating characteristic partial area index for highly sensitive diagnostic tests Radiology 1996 201 745 750 8939225 10.1148/radiology.201.3.8939225

[B9] Streiner DL Cairney J What's under the ROC? An introduction to receiver operating characteristics curves Canadian Journal of Psychiatry Revue Canadienne De Psychiatrie 2007 52 121 128 17375868 10.1177/070674370705200210

[B10] Stephan C Wesseling S Schink T Jung K Comparison of Eight Computer Programs for Receiver-Operating Characteristic Analysis Clin Chem 2003 49 433 439 10.1373/49.3.433 12600955

[B11] R Development Core Team R: A Language and Environment for Statistical Computing 2010 Vienna, Austria: R Foundation for Statistical Computing

[B12] Sing T Sander O Beerenwinkel N Lengauer T ROCR: visualizing classifier performance in R Bioinformatics 2005 21 3940 3941 10.1093/bioinformatics/bti623 16096348

[B13] NCAR verification: Forecast verification utilities v. 1.31 http://CRAN.R-project.org/package=verification

[B14] Carey V Redestig H ROC: utilities for ROC, with uarray focus, v. 1.24.0 http://www.bioconductor.org

[B15] Pepe M Longton G Janes H Estimation and Comparison of Receiver Operating Characteristic Curves The Stata journal 2009 9 1 20161343 PMC2774909

[B16] Hanley JA McNeil BJ A method of comparing the areas under receiver operating characteristic curves derived from the same cases Radiology 1983 148 839 843 6878708 10.1148/radiology.148.3.6878708

[B17] DeLong ER DeLong DM Clarke-Pearson DL Comparing the Areas under Two or More Correlated Receiver Operating Characteristic Curves: A Nonparametric Approach Biometrics 1988 44 837 845 10.2307/2531595 3203132

[B18] Bandos AI Rockette HE Gur D A permutation test sensitive to differences in areas for comparing ROC curves from a paired design Stat Med 2005 24 2873 2893 10.1002/sim.2149 16134144

[B19] Braun TM Alonzo TA A modified sign test for comparing paired ROC curves Biostat 2008 9 364 372 10.1093/biostatistics/kxm036 17925302

[B20] Venkatraman ES Begg CB A distribution-free procedure for comparing receiver operating characteristic curves from a paired experiment Biometrika 1996 83 835 848 10.1093/biomet/83.4.835

[B21] Bandos AI Rockette HE Gur D A Permutation Test for Comparing ROC Curves in Multireader Studies: A Multi-reader ROC, Permutation Test Acad Radiol 2006 13 414 420 10.1016/j.acra.2005.12.012 16554220

[B22] Moise A Clement B Raissis M A test for crossing receiver operating characteristic (roc) curves Communications in Statistics - Theory and Methods 1988 17 1985 2003 10.1080/03610928808829727

[B23] Venkatraman ES A Permutation Test to Compare Receiver Operating Characteristic Curves Biometrics 2000 56 1134 1138 10.1111/j.0006-341X.2000.01134.x 11129471

[B24] Campbell G Advances in statistical methodology for the evaluation of diagnostic and laboratory tests Stat Med 1994 13 499 508 10.1002/sim.4780130513 8023031

[B25] Wickham H plyr: Tools for splitting, applying and combining data v. 1.4 http://CRAN.R-project.org/package=plyr

[B26] Carpenter J Bithell J Bootstrap confidence intervals: when, which, what? A practical guide for medical statisticians Stat Med 2000 19 1141 1164 10.1002/(SICI)1097-0258(20000515)19:9<1141::AID-SIM479>3.0.CO;2-F 10797513

[B27] Metz CE Herman BA Shen JH Maximum likelihood estimation of receiver operating characteristic (ROC) curves from continuously-distributed data Stat Med 1998 17 1033 1053 10.1002/(SICI)1097-0258(19980515)17:9<1033::AID-SIM784>3.0.CO;2-Z 9612889

[B28] Hanley JA The robustness of the "binormal" assumptions used in fitting ROC curves Med Decis Making 1988 8 197 203 10.1177/0272989X8800800308 3398748

[B29] Zou KH Hall WJ Shapiro DE Smooth non-parametric receiver operating characteristic (ROC) curves for continuous diagnostic tests Stat Med 1997 16 2143 2156 10.1002/(SICI)1097-0258(19971015)16:19<2143::AID-SIM655>3.0.CO;2-3 9330425

[B30] Venables WN Ripley BD Modern Applied Statistics with S 2002 Fourth New York: Springer

[B31] Turck N Vutskits L Sanchez-Pena P Robin X Hainard A Gex-Fabry M Fouda C Bassem H Mueller M Lisacek F A multiparameter panel method for outcome prediction following aneurysmal subarachnoid hemorrhage Intensive Care Med 2010 36 107 115 10.1007/s00134-009-1641-y 19760205

[B32] Ewens WJ Grant GR Statistics (i): An Introduction to Statistical Inference Statistical methods in bioinformatics 2005 New York: Springer-Verlag

