# Supplementary material for: pROC: an open-source package for R and S+ to analyze and compare ROC curves
Source: BMC Bioinformatics. 2011 Mar 17;12:77. doi: 10.1186/1471-2105-12-77 (PMC3068975; doi:10.1186/1471-2105-12-77)
Supplement: Additional file 1 — Assessment of the ROC comparison tests. We evaluate the uniformity of the tests under the null hypothesis (ROC curves are not different), and the correlation between the different tests. [file 1471-2105-12-77-S1.PDF]

Supplementary text for *pROC: an open-source package for R and S+ to analyze and compare ROC curves*

Assessment of the ROC comparison tests

To ensure that our implementations of the three statistical tests are correct, and to evaluate the correlation between them, we generated 600 p-values for each test under the null hypothesis (ROC curves are not different) by randomly switching the class labels of the 141 aSAH patients. For each null hypothesis, DeLong, Venkatraman (with 10000 permutations) and bootstrap (with 10, 100, 1000 and 10000 replicates) tests were performed with a paired and unpaired setup.

We first assessed the existence of a systematic bias towards high or low p-values. Additional File 2 shows that the paired tests do not deviate from uniformity under the null hypothesis (One-sample Kolmogorov-Smirnov test, $p = 0.99$ for DeLong’s test, $p = 0.96$ for bootstrap test and $p = 0.32$ for Venkatraman’s test). However paired test are slightly biased towards higher p-values (One-sample Kolmogorov-Smirnov test, $p = 0.02$ for DeLong’s test, $p = 0.03$ for bootstrap, $p = 0.03$ for Venkatraman).

Next, we tested the relationship between DeLong and bootstrap tests. Both tests determine differences in AUCs and should produce similar results. Indeed, Additional File 3 shows that with enough bootstrap replicates, the bootstrap test converges to the values of DeLong’s test. Note that DeLong’s test is a deterministic test and thus is not subject to variations when repeated tests are performed on the same data. Spearman's rank correlation ρ is above 0.99 for all tests with 100 or more bootstrap replicates. For paired p-values lower than 0.1, the absolute difference between DeLong and bootstrap p-values obtained after 10000 replicates was lower than 0.005 in 95% of the tests. The 95% range of the differences increased to 0.011 and 0.03 for 1000 and 100 replicates respectively. For unpaired p-values, the same trend was observed with 95% of the differences within 0.007, 0.013 and 0.03 for 10000, 1000 and 100 replicates respectively. Therefore, the second decimal of the p-value is measured accurately with 10000 bootstrap replicates, but not with 1000 or less replicates.

Finally, we looked at the association between Delong and Venkatraman’s tests. In contrast with the bootstrap test, Venkatraman’s test does not estimate the AUC but rather the shape of the ROC curve. Thus, we expect a lower correlation than with bootstrap, as two ROC curves with a different shape can have a similar or identical AUC value. Indeed, Additional File 4 shows a much lower correlation than that observed in Additional File 3 with bootstrap. Note that the figure is asymmetric: similar AUCs may have different shapes, but it is less likely that similar shapes would have different AUCs.
