# Supplementary material for: pROC: an open-source package for R and S+ to analyze and compare ROC curves
Source: BMC Bioinformatics. 2011 Mar 17;12:77. doi: 10.1186/1471-2105-12-77 (PMC3068975; doi:10.1186/1471-2105-12-77)
Supplement: Additional file 2 — Histograms of the frequency of 600 test p-values under the null hypothesis (ROC curves are not different). A: DeLong's paired test, B: DeLong's unpaired test, C: bootstrap paired test (with 10000 replicates), D: bootstrap unpaired test (with 10000 replicates) and E: Venkatraman's test (with 10000 permutations). [file 1471-2105-12-77-S2.PDF]

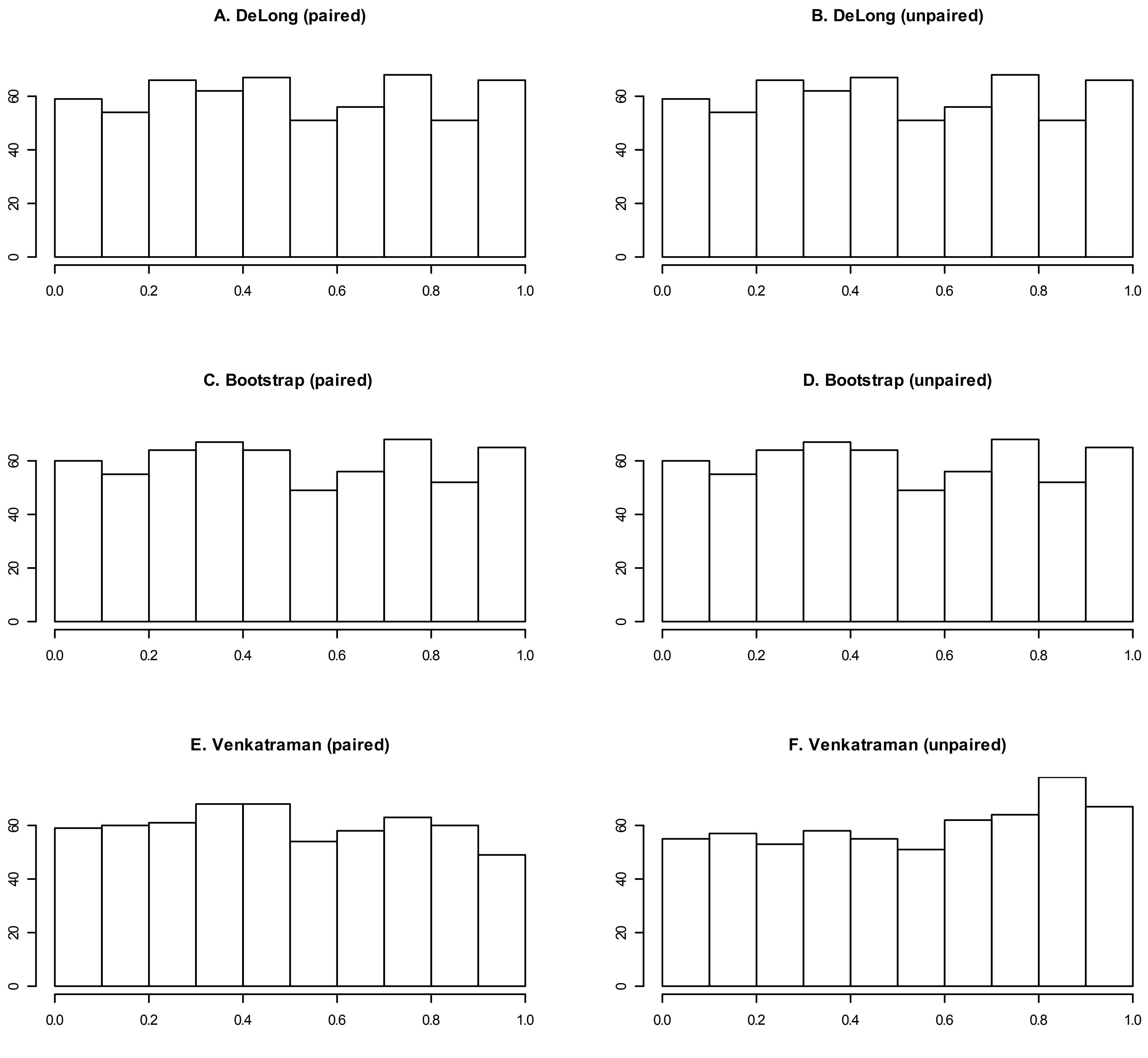
A. DeLong (paired)
B. DeLong (unpaired)
C. Bootstrap (paired)
D. Bootstrap (unpaired)
E. Venkatraman (paired)
F. Venkatraman (unpaired)
0
20
40
60
0.0
0.2
0.4
0.6
0.8
1.0
