# Supplementary figures and images for: pROC: an open-source package for R and S+ to analyze and compare ROC curves
Source: BMC Bioinformatics. 2011 Mar 17;12:77. doi: 10.1186/1471-2105-12-77 (PMC3068975; doi:10.1186/1471-2105-12-77)

**A. 10**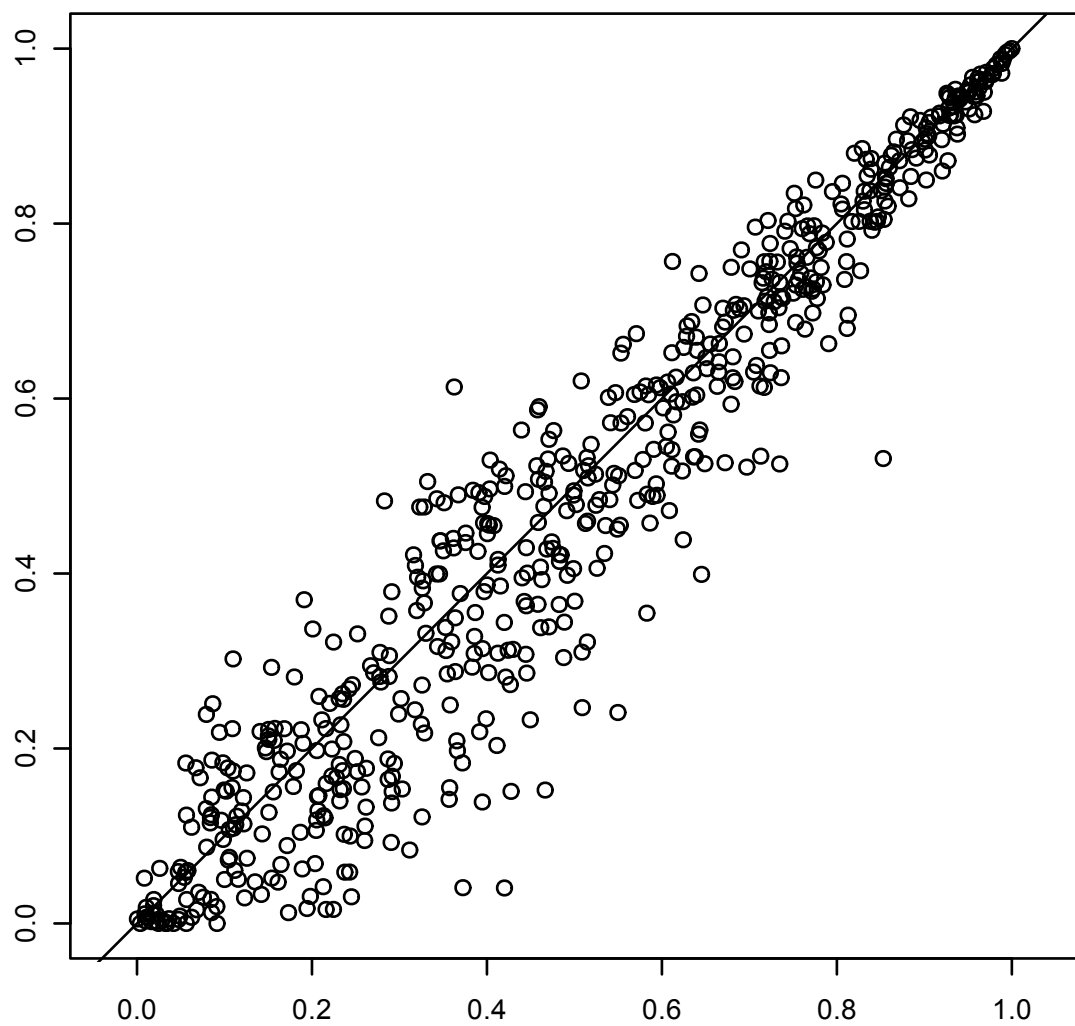**B. 100**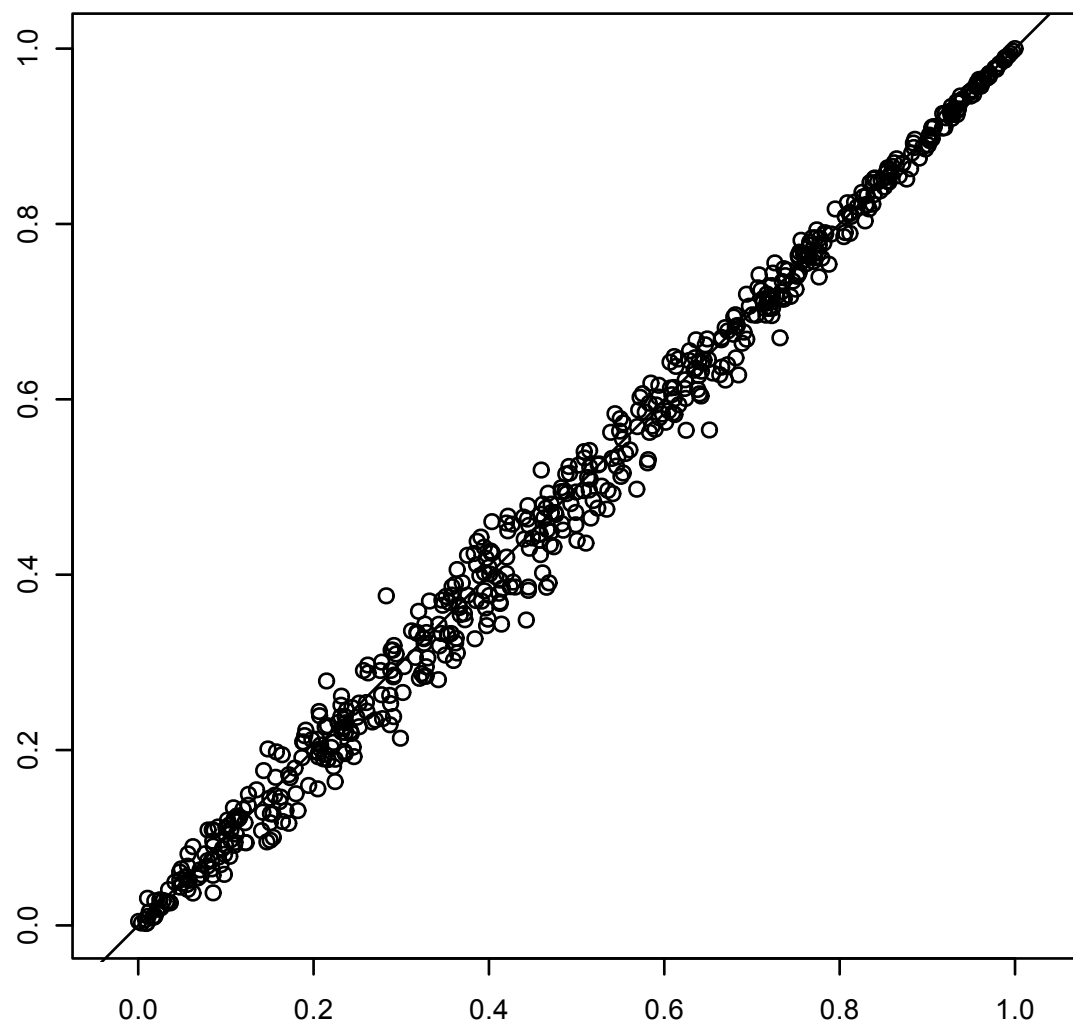**C. 1000**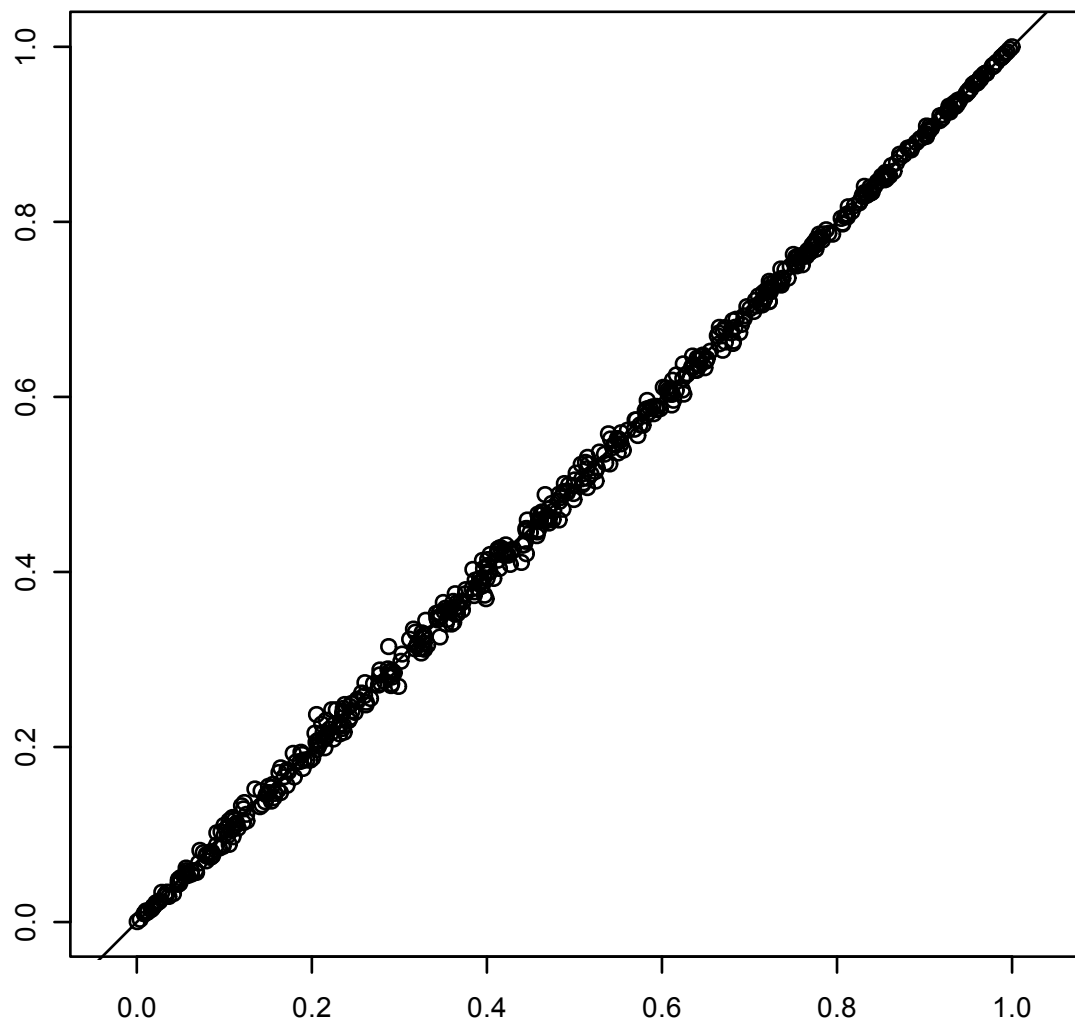**D. 10000**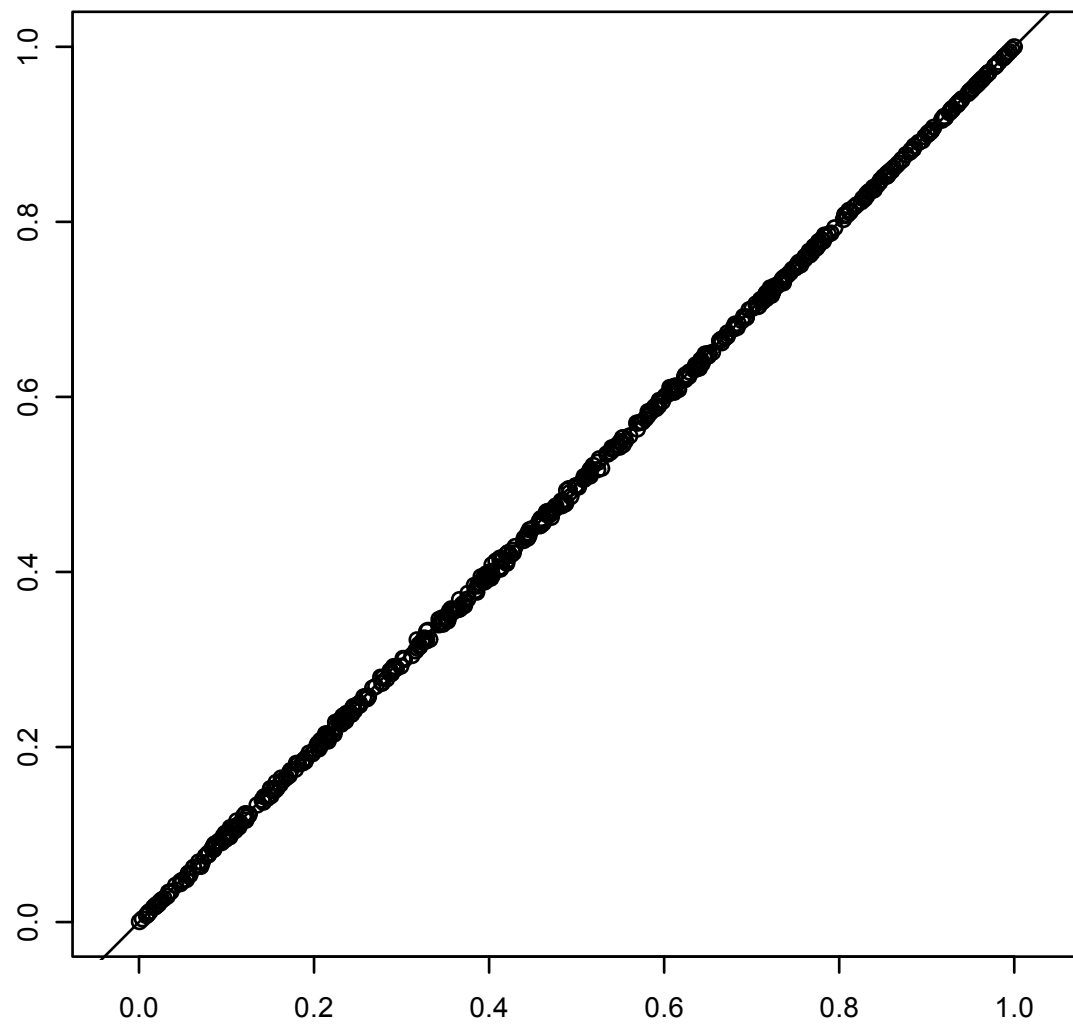

DeLong

Supplement: Additional file 3 — Correlations between DeLong and bootstrap paired tests. X axis: DeLong's test; Y-axis: bootstrap test with number of bootstrap replicates. A: 10, B: 100, C: 1000 and D: 10000. [file 1471-2105-12-77-S3.PDF]

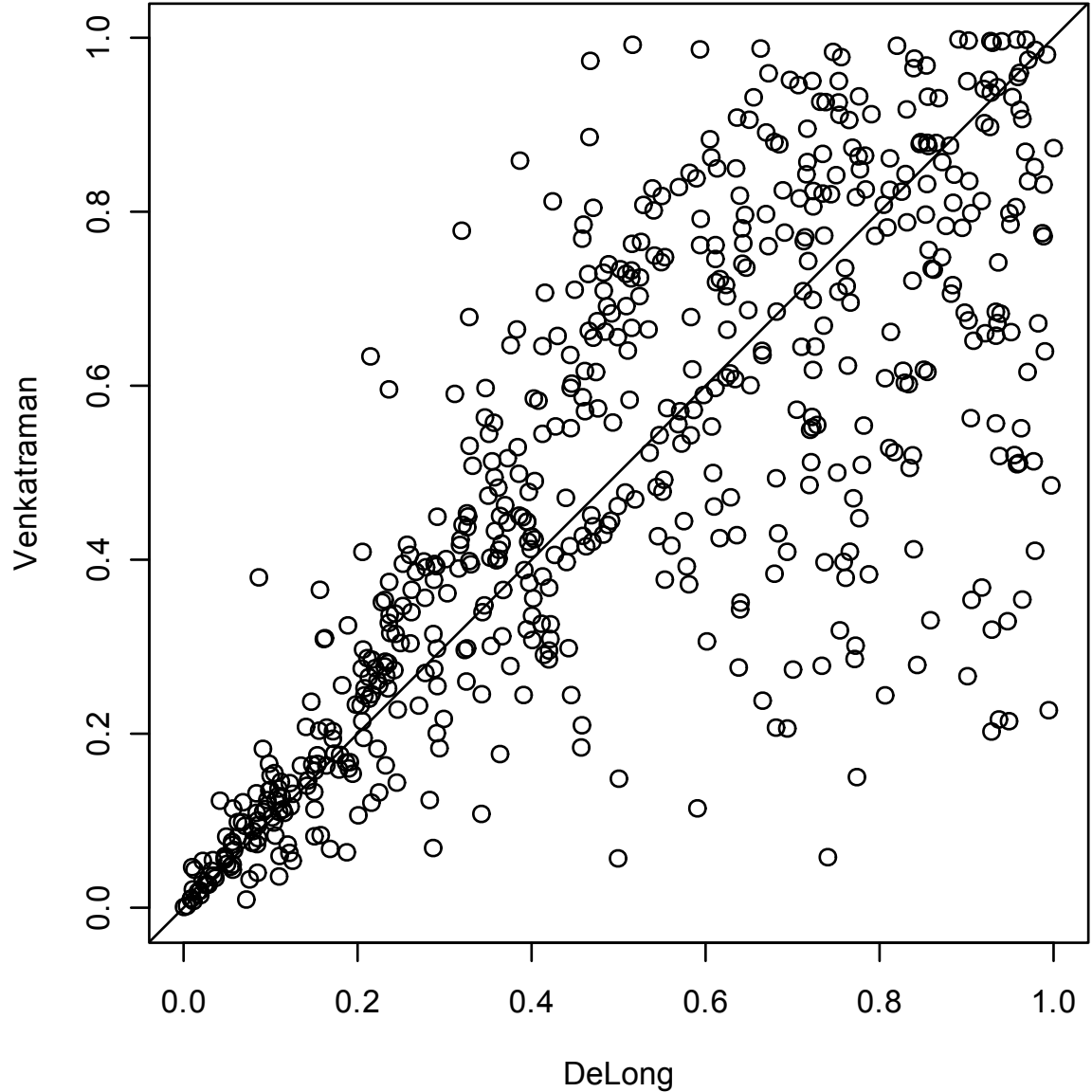

Supplement: Additional file 4 — Correlation between DeLong and Venkatraman's test. X axis: DeLong's test; Y-axis: Venkatraman's test with 10000 permutations. [file 1471-2105-12-77-S4.PDF]

markers: ndka

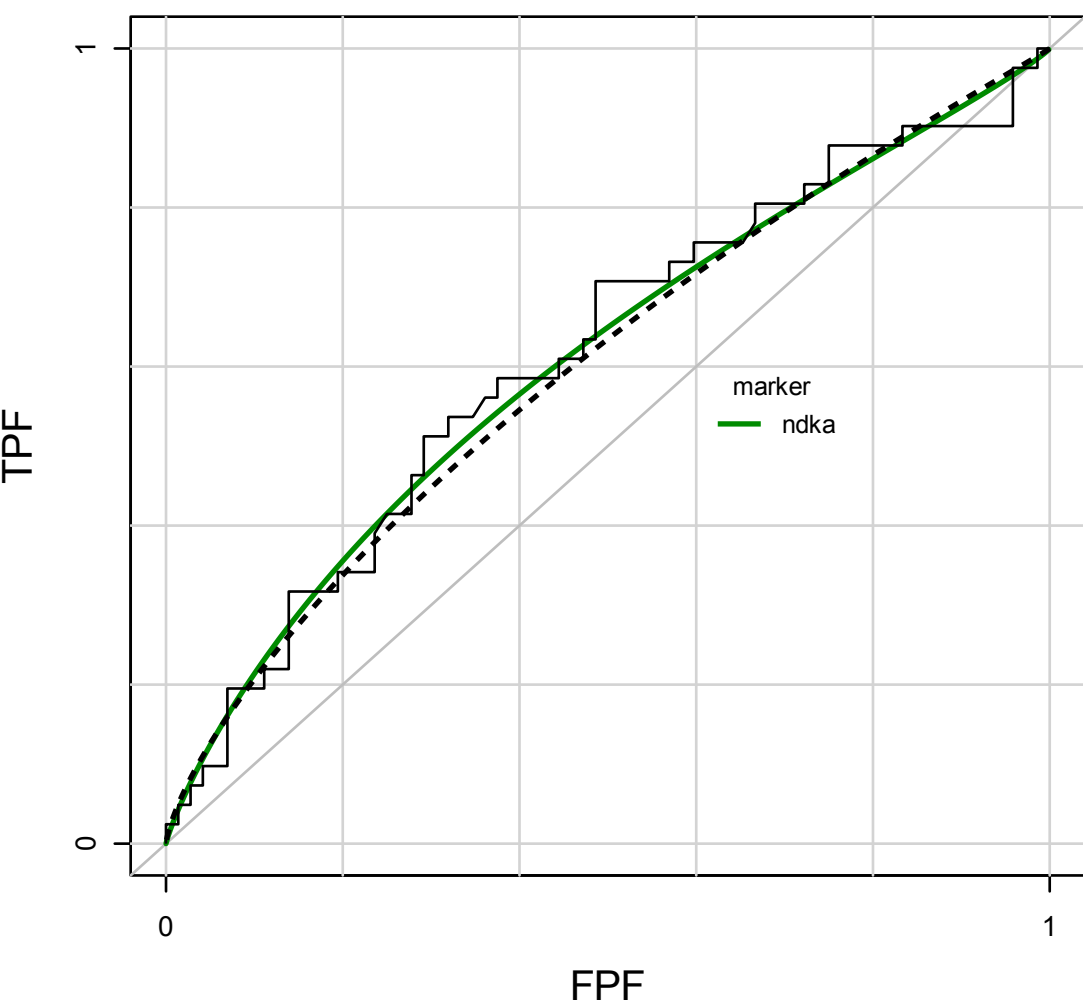

markers: wfns

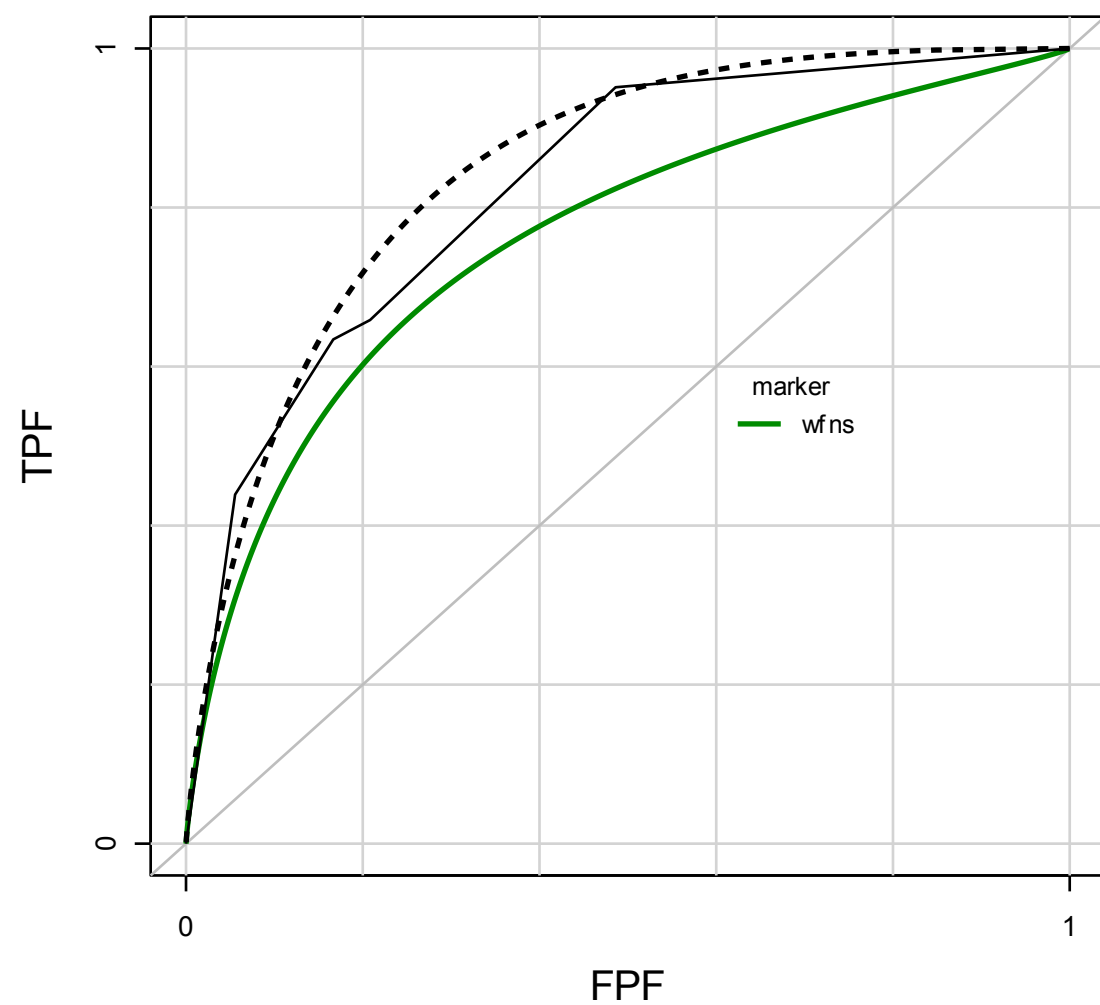

markers: s100b

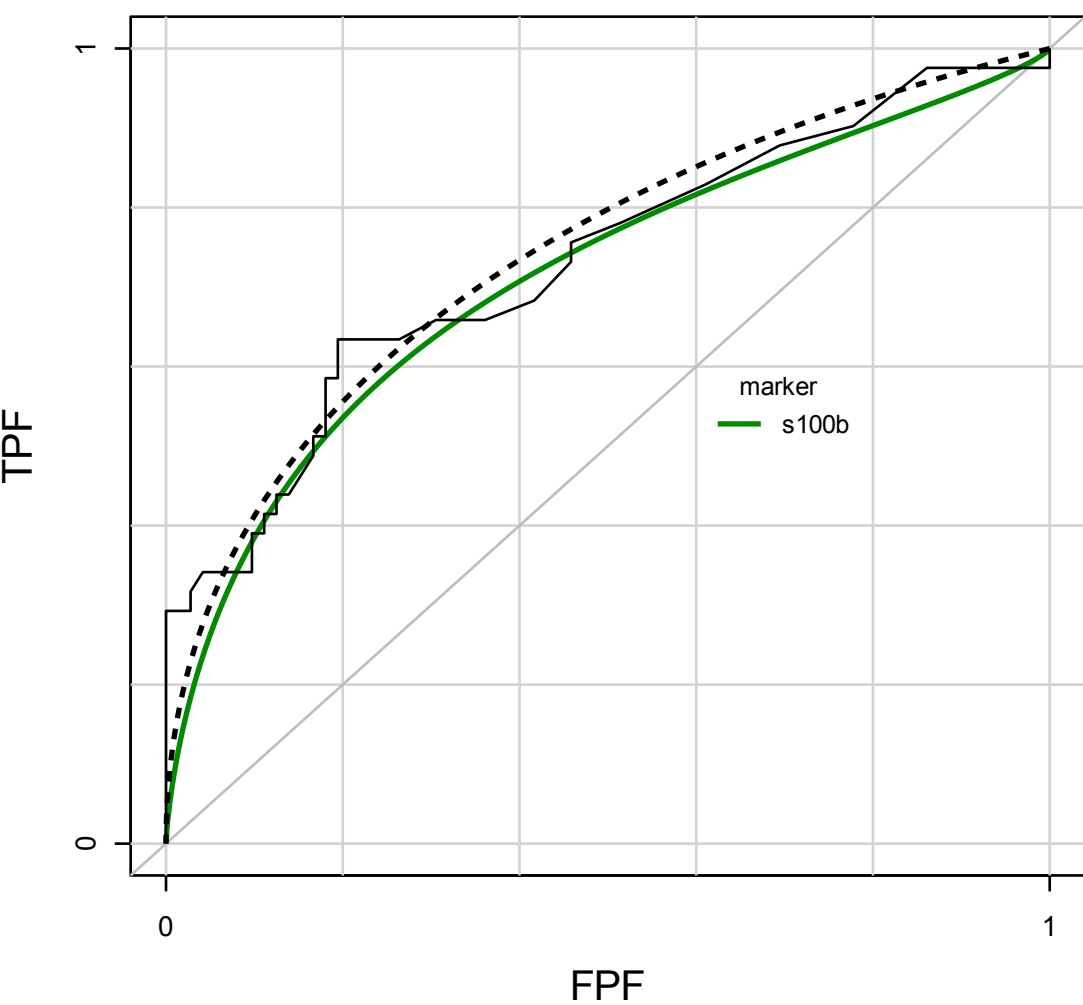

markers: age

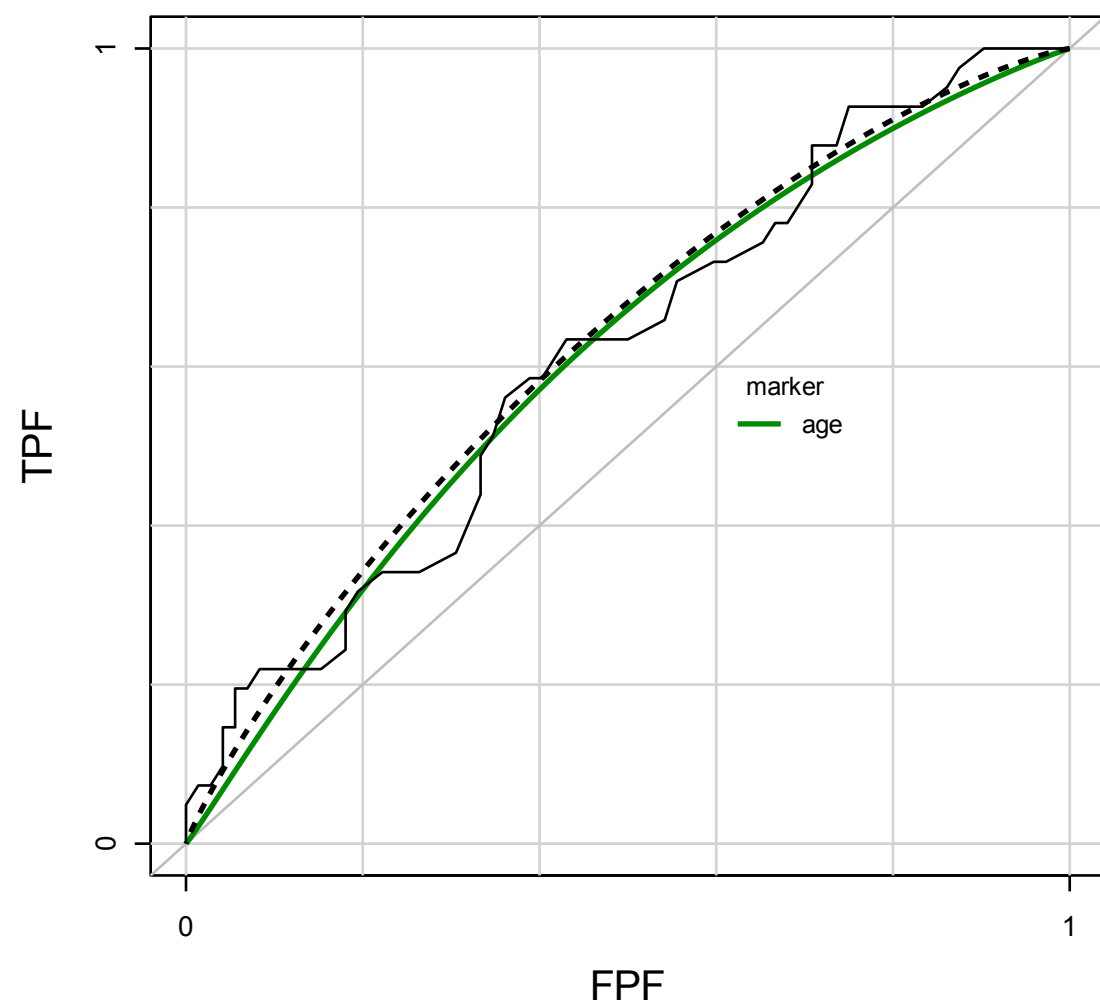

Supplement: Additional file 5 — Binormal smoothing. Binormal smoothing with pcvsuite (green, solid) and pROC (black, dashed). [file 1471-2105-12-77-S5.PDF]
